# Ultrasound-based “CEUS-Bosniak”classification for cystic renal lesions: an 8-year clinical experience

**DOI:** 10.1007/s00345-022-04094-0

**Published:** 2022-08-20

**Authors:** Elena Herms, Gregor Weirich, Tobias Maurer, Stefan Wagenpfeil, Stephanie Preuss, Andreas Sauter, Matthias Heck, Anita Gärtner, Katharina Hauner, Michael Autenrieth, Hubert P. Kübler, Konstantin Holzapfel, Ulrike Schwarz-Boeger, Uwe Heemann, Julia Slotta-Huspenina, Konrad Friedrich Stock

**Affiliations:** 1 Department of Nephrology, University Hospital MRI-TUM (München Rechts Der Isar), Munich, Germany; 2 Institute of Pathology, University Hospital MRI-TUM (München Rechts Der Isar), Munich, Germany; 3 Department of Anesthesia, Freising University Hospital, Freising, Germany; 4 Department of Urology, University Hospital MRI-TUM (München Rechts Der Isar), Munich, Germany; 5 Department of Urology and Martini-Klinik, University Hospital Hamburg-Eppendorf, Hamburg, Germany; 6 Department of Urology, University Hospital Würzburg, Würzburg, Germany; 7 Department of Radiology, Hospital Landshut-Achdorf, Landshut, Germany; 8 Department of Radiology, University Hospital MRI-TUM (München Rechts Der Isar), Munich, Germany; 9 Medical Controlling, University Hospital MRI-TUM (München Rechts Der Isar), Munich, Germany; 10 Institute of Medical Biometry, Epidemiology and Medical Informatics (IMBEI), Saarland University, Campus Homburg, Homburg, Germany

**Keywords:** Complex renal cyst, Bosniak classification, CEUS-Bosniak, Contrast-enhanced ultrasound, CEUS, Cystic renal masses

## Abstract

**Purpose:**

Renal cysts comprise benign and malignant entities. Risk assessment profits from CT/MRI imaging using the Bosniak classification. While Bosniak-IIF, -III, and -IV cover complex cyst variants, Bosniak-IIF and -III stand out due to notorious overestimation. Contrast-enhanced ultrasound (CEUS) is promising to overcome this deficit but warrants standardization. This study addresses the benefits of a combined CEUS and CT/MRI evaluation of renal cysts. The study provides a realistic account of kidney tumor boards' intricacies in trying to validate renal cysts.

**Methods:**

247 patients were examined over 8 years. CEUS lesions were graded according to CEUS-Bosniak (IIF, III, IV). 55 lesions were resected, CEUS-Bosniak- and CT/MRI-Bosniak-classification were correlated with histopathological diagnosis. Interobserver agreement between the classifications was evaluated statistically. 105 lesions were followed by ultrasound, and change in CEUS-Bosniak-types and lesion size were documented.

**Results:**

146 patients (156 lesions) were included. CEUS classified 67 lesions as CEUS-Bosniak-IIF, 44 as CEUS-Bosniak-III, and 45 as CEUS-Bosniak-IV. Histopathology of 55 resected lesions revealed benign cysts in all CEUS-Bosniak-IIF lesions (2/2), 40% of CEUS-Bosniak-III and 8% of CEUS-Bosniak-IV, whereas malignancy was uncovered in 60% of CEUS-Bosniak-III and 92% of CEUS-Bosniak-IV. Overall, CEUS-Bosniak-types matched CT/MRI-Bosniak types in 58% (fair agreement, κ = 0.28). CEUS-Bosniak resulted in higher stages than CT/MRI-Bosniak (40%). Ultrasound follow-up of 105 lesions detected no relevant differences between CEUS-Bosniak-types concerning cysts size. 99% of lesions showed the same CEUS-Bosniak-type.

**Conclusion:**

The CEUS-Bosniak classification is an essential tool in clinical practice to differentiate and monitor renal cystic lesions and empowers diagnostic work-up and patient care.

## Introduction

Medical imaging represents an indispensable tool of modern diagnostics. Pillars of this non-invasive approach are computed tomography (CT), magnetic resonance imaging (MRI), and multimodal ultrasound exams (US), each of which come along with methodological advantages and disadvantages with respect to specific technical potentials and diagnostic needs. The application of medical imaging has not only helped to better define and monitor diseases, but has also broadened our knowledge of incidentalomas like renal cysts. Renal cysts develop in the context of advanced age, male gender, and impaired renal function. [1, 2] Efforts to ascribe detailed imaging features of renal cysts resulted in the Bosniak classification for CT and contrast enhanced ultrasound (CEUS). Both systems differentiate between simple and complex cysts. [3, 4] A CT-based European study with 617 patients showed a prevalence of 41% for simple renal cysts [5], whereas a larger ultrasound-based Japanese study (17 914 individuals) showed a prevalence of 9.9%. [1] Risk factors for malignancy of complex renal cysts are male gender, younger age (< 50 years), and cyst size (> 2 cm) [2], as well as history of renal cell carcinoma, body mass index, and African American race. [6] To classify renal cysts, the CT-based Bosniak classification was established. The risk of malignancy increases with higher CT-Bosniak types. [7] In 2019, Silverman et al. proposed an integration of MRI [8]. CT-Bosniak-I and -II lesions are considered benign. [7] Whereas the majority of CT-Bosniak-IIF cysts run a benign clinical course [9, 10], CT-Bosniak-III holds a substantial number of risky lesions (43–79%) [9, 11, 12], and CT-Bosniak-IV lesions are mainly malignant (85–100%) [2, 11–13]. It is, therefore, evident that CT-Bosniak-III warrants finetuning of discriminators of malignancy. Ultrasound may help to solve this dilemma. Ultrasound (B-mode, Color Doppler) is widely available. CEUS is mandatory in the differential diagnosis of "non-simple" cysts, because it shows real-time blood flow and microvascularization. Recently, a European proposal for CEUS-adapted Bosniak categorization was published. [14] CEUS profits from the detailed perfusion information of complex lesions and will, therefore, help to guide therapy. This study focused on the histopathologic findings of CEUS-Bosniak-IIF, -III, and -IV lesions. CEUS-Bosniak classification was compared to current standard clinical imaging (CE-CT/MRI). Furthermore, size variation of CEUS-Bosniak-IIF, -III, and -IV cysts was followed-up with respect to its discriminatory power.

## Materials and methods

### Study population

CEUS-Bosniak lesions were identified by a data base query in the clinical information system of the university hospital “Klinikum rechts der Isar”, Munich, Germany. The query spanned an 8-year period (08/2013–08/2021). Inclusion criteria were diagnosis of CEUS-Bosniak-IIF, -III and -IV, lesion monitoring (at least one ultrasound follow-up scan), or lesion resection (with histopathological report). Excluded were lesions in transplanted kidneys. Ethical approval was granted by the Ethics committee of the Technical University of Munich (TUM) (40/21 S-EB).

### Imaging

#### CEUS

During the study period, four high-end ultrasound devices were used: Siemens-Acuson S 2000, Siemens-Acuson Sequoia 512, Siemens-Acuson Sequoia (Siemens Healthineers, Mountain View, USA), and Logic E9 (GE Healthcare, Chicago, USA) with convex and linear probes. Ultrasound modalities included B-mode, Doppler, and CEUS, where sulfur hexafluoride ultrasound contrast agent (Sonovue®, Bracco Imaging, Milan) was used. Lesions were classified according to the CEUS-Bosniak classification. A change in size was defined as a deviation of more than 10% in diameter. For lesions undergoing follow-up, diameters were determined during the first and last exam.

#### CT/MRI

Inhouse and external imaging data were analyzed using PACS, evaluated by a blinded kidney-experienced radiologist consultant, and classified according to CT-/MRI-Bosniak classification.

### Histopathology

Histopathology reports of all study patients with a history of partial or total nephrectomy were retrieved from the archives of the Institute of Pathology of the TUM. Selected cases were reclassified according to the current WHO classification. [15] Histopathological examinations were carried out by board-certified pathologists with expertise in urologic pathology.

### Statistics

Statistics applied SPSS v. 27 (IBM SPSS Statistics for Mac, Armonk, NY, IBM Corp). Continuous variables were specified as median with interquartile range (IQR). Frequencies and proportions were used to express categorical variables. The Cohen’s kappa index was calculated to assess the agreement between the CEUS- and CT/MRI-Bosniak classification. A threshold of *p* < 0.05 (two-sided) was considered significant.

## Results

**Fig. 1:**
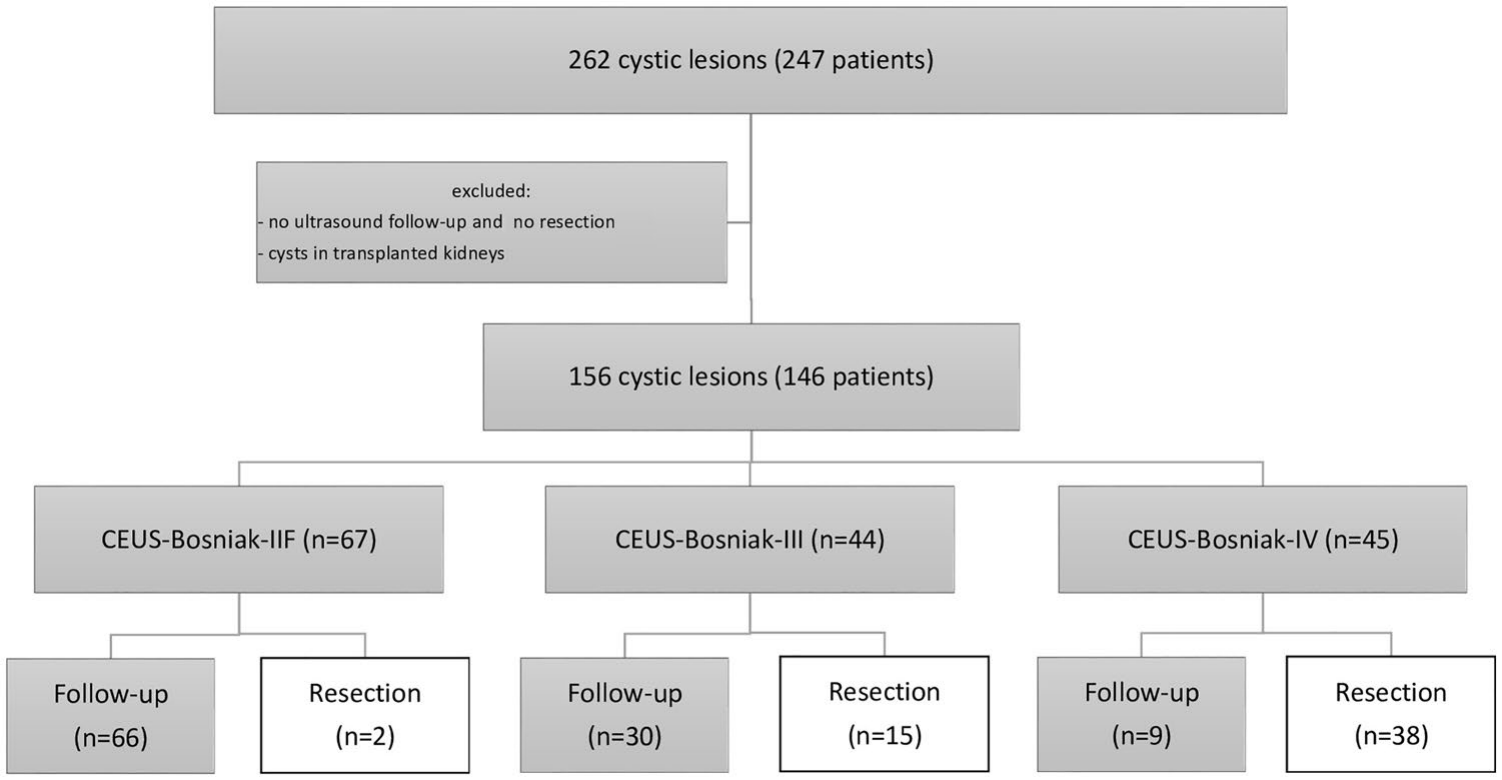
Study cohort

Data query retrieved 247 patients with 262 CEUS-Bosniak-IIF/-III/-IV lesions. Of these, 156 matched the inclusion criteria (146 patients, median age 59 years, 29–81 years; 97 male, 49 female). 105/156 lesions (67.3%) had been followed-up by ultrasound, and 55/156 lesions (35.2%) were resected and classified by histopathology (Fig. 1). CT/MRI was evaluated in 52/55 resected lesions and classified according to CT/MRI-Bosniak.

### Correlation of CEUS-Bosniak with pathomorphology

All resected lesions contained grossly visible cystoid areas. Pathology appraisal found different kinds of cyst-like cavities: (a) true epithelial cysts (uni-/multilocular) containing watery fluid/blood with or without adjacent solid areas composed of benign fibroblasts or malignant epithelia (renal cell carcinoma, RCC), (b) pseudocysts composed of a necrotic core and a rim of vital malignant epithelia (clear cell RCC, papillary RCC, chromophobe RCC, unclassified RCC).

Whereas the two CEUS-Bosniak-IIF lesions (*n* = 2) were diagnosed as simple renal cysts, the categories of CEUS-Bosniak-III (*n* = 15) and CEUS-Bosniak-IV (*n* = 38) included benign and malignant lesions. CEUS-Bosniak-III lesions were diagnosed as benign in 40% and as malignant in 60%. CEUS-Bosniak-IV lesions were benign in 7.9% and malignant in 92.1%. Diagnoses are summarized in Table 1. Simple renal cysts, when classified as CEUS-Bosniak-III, showed extensive bleeding, regressive changes and bridge-forming fibrosis. The new WHO-entity of multilocular cystic renal neoplasia of low malignant potential (MCRNLMP, formerly called multilocular cystic renal cell carcinoma) was classified by CEUS as CEUS-Bosniak-III (*n* = 3) and CEUS-Bosniak-IV (*n* = 4).

**Table 1:** Histopathological diagnoses of CEUS-Bosniak-IIF, -III and -IV lesions

CEUS-Bosniak category	Histopathological diagnosis
CEUS-Bosniak-IIF (*n* = 2)	Simple renal cyst (2/2)
CEUS-Bosniak-III (*n* = 15)	Simple renal cyst (4/15; 26.7%) Cystic nephroma/ mixed epithelial stroma tumor (MEST) (2/15; 13.3%) Multilocular cystic renal neoplasm of low malignant potential (3/15; 20%) Clear cell renal cell carcinoma (ccRCC) (6/15; 40%)
CEUS-Bosniak-IV (*n* = 38)	Simple renal cyst (2/38; 5.3%) Cystic nephroma/mixed epithelial stroma tumor (MEST) (1/38; 2.6%) Multilocular cystic renal neoplasm of low malignant potential (4/38; 10.5%) Clear cell renal cell carcinoma (ccRCC) (17/38; 44.7%) Papillary renal cell carcinoma (PRCC) (10/38; 26.3%) Chromophobe renal cell carcinoma (ChRCC) (2/38; 5.2%) Unclassified renal cell carcinoma (2/38; 5.2%)

### Correlation of CT/MRI-Bosniak with histopathology

CT-/MRI-images were available for 52/55 resected lesions. CT/MRI-Bosniak was I/II in 2 lesions (3.8%), IIF in 5 (9.6%), III in 24 (46.1%), and IV in 21 lesions (40.4%). Whereas CT/MRI-Bosniak-I/II were solely benign (simple cysts, 2/2), CT/MRI-Bosniak-IIF, -III and -IV included benign and malignant diagnoses. CT/MRI-Bosniak-IIF corresponded to benignity in 1/5 (simple renal cyst, *n* = 1), malignancy in 4/5 (MCRNLMP, *n* = 1; PRCC, n = 2; ChRCC, *n* = 1). CT/MRI-Bosniak-III lesions were in line with benignity in 5/24 (simple renal cyst, *n* = 2; MEST, *n* = 3), and malignancy in 19/24 (MCRNLMP, *n* = 6; clear cell RCC, *n* = 12; papillary RCC, *n* = 1). CT/MRI-Bosniak-IV lesions corresponded to benignity in 1/21 (simple renal cysts, *n* = 1), and to malignancy in 20/21 (ccRCC, *n* = 10; PRCC, *n* = 7; ChRCC, *n* = 3).

### Agreement of CEUS and CT/MRI imaging

Matches: CEUS-Bosniak classification matched with CT/MRI-Bosniak in 57.7% (30/52). Statistics found a quite fair agreement between the two classifications (κ = 0.28, *p* = 0.004).

### Mismatches

**Fig. 2:**
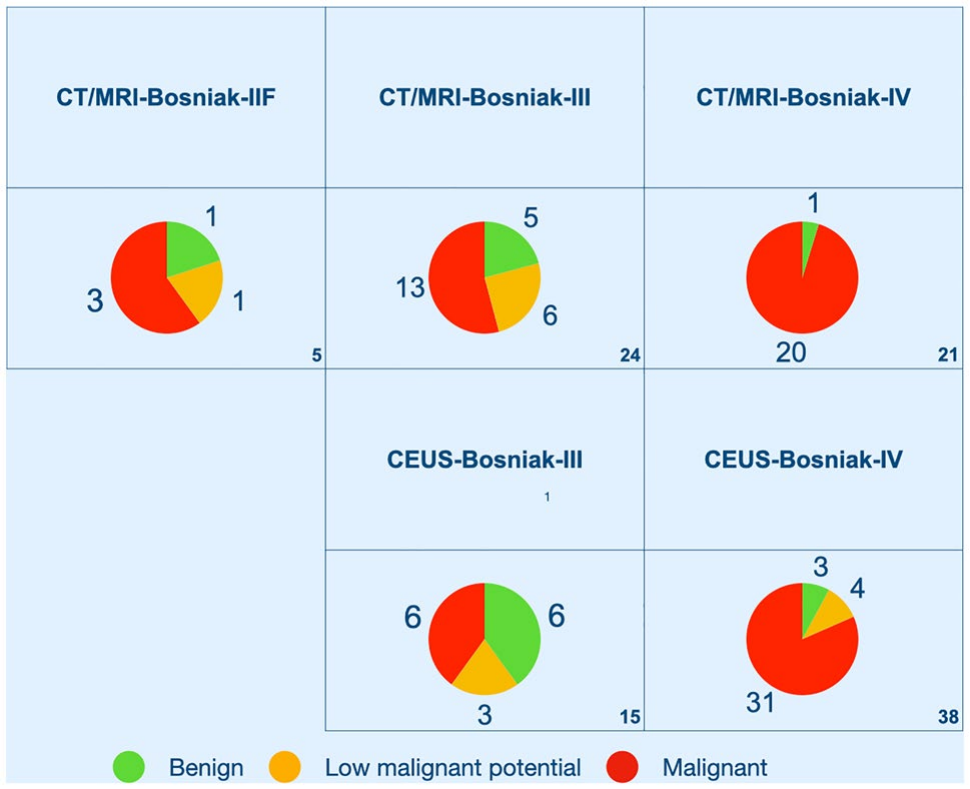
Comparison of CEUS-Bosniak classification and CT/MRI-Bosniak classification in resected cysts. Pie charts show the percentage of patients with benign (green), low malignant (yellow), and malignant findings (red); numbers (bottom right) indicate the number of patients

Mismatches were noted in 42.3% (22/52). In 40.4% (21/52) CEUS-Bosniak resulted in higher stages than CT/MRI-Bosniak, the opposite was the case in 1.9% (1/52). Lesions, where CEUS-Bosniak scored higher than CT/MRI-Bosniak were: two CT/MRI-Bosniak-I/II lesions scored as CEUS-Bosniak-III, eventually diagnosed as benign simple renal cysts. One CT/MRI-Bosniak-IIF lesion scored as CEUS-Bosniak-III, pathology revealed a MCRNLMP. Three CT/MRI-Bosniak-IIF lesions scored as CEUS-Bosniak-IV, pathology detected RCC (ChRCC, PRCC, ccRCC). Fifteen CT/MRI-Bosniak-III lesions scored as CEUS-Bosniak-IV. Pathology detected 2 benign lesions (simple renal cyst, MEST) and 13 malignant tumors (8 ccRCC, 1 PRCC and 4 MCRNLMP). CT/MRI- and CEUS-Bosniak-III/-IV classes with correspondent benign, semi-malignant and malignant diagnoses are displayed in Fig. 2. Between benign, semi-malignant and malignant findings in CT/MRI-, and CEUS-Bosniak categories, we detected overlaps, except for CT/MRI-Bosniak-IV, that were almost malignant (95.2%).

### Ultrasound follow-up of CEUS-Bosniak-IIF, -III and -IV lesions

66 CEUS-Bosniak-IIF, 30 CEUS-Bosniak-III, and nine CEUS-Bosniak-IV lesions were followed-up. The latter had not been resected because of comorbidities or patient denial. CEUS-Bosniak-IIF lesions were monitored over a median period of 2.1 years (range 0.3–9.4). Most CEUS-Bosniak-IIF lesions remained stable in size (42.4%; 28/66), whereas an increase in size was observed in 27.3% (18/66) with maximum growth of 24 mm/year. A size reduction was seen in 30.3% (20/66) with maximum diminution of 43 mm/year. No CEUS-Bosniak-IIF lesion entailed an upgrade. CEUS-Bosniak-III lesions were followed over a median period of 1.4 years (range 0.5–7.7). 20% of the lesions (6/30) increased (up to 10 mm/year), 23.3% (7/30) decreased (up to 10 mm/year), while most of CEUS-Bosniak-III lesions remained unchanged (56.7%; 17/30). None of the CEUS-Bosniak-III lesions were upgraded, one CEUS-Bosniak-III was downgraded to CEUS-Bosniak-IIF. CEUS-Bosniak-IV lesions were observed over a median period of 1.1 years (range 0.7–4.8). An increase in size was noted in 44.4% (4/9) with maximum growth of 4 mm/year and a decrease was seen in 22.2% (2/9) with up to 47 mm/year. 33.3% (3/9) of CEUS-Bosniak-IV lesions remained unchanged. CEUS-Bosniak-IV lesions were not downgraded.

## Discussion

Diagnosis and therapy of renal cysts have experienced continuous improvements in the era of imaging. CT/MRI-based imaging helped to develop diagnostic criteria for the discrimination of benign and malignant cysts. Categorisation of CT/MRI findings was introduced by Bosniak, discriminating benign (type I–II) from potentially malignant (type IIF–III) and malignant lesions (type IV). [7] This system being in use for almost 40 years has shown its potential for accurate detection of malignancy (high sensitivity), with the drawback that Bosniak-IIF and -III include the whole spectrum of benign and malignant lesions in a substantial number. Especially the Bosniak-III category is, therefore, of limited help for therapy planning. [16] Currently, management recommendations for CT-Bosniak-III lesions discuss active surveillance. [17, 18] In line with this, studies showed high survival for patients with complex renal cysts undergoing active surveillance. [17].

The advent of ultrasound diagnostics gave birth to a novel imaging modality with a capability of spatial lesion resolution. Combined with contrast enhancement to register blood flow, a more accurate imaging of cystic lesions is now possible. Currently, CEUS is mandatory for the characterization of "non-simple" renal cysts. CEUS excels in tracking malignancy, even in lesions deemed innocuous by CT/MRI, because CEUS can detect minute blood flow in hypovascular cancer lesions. [19] This advantage is evident in PRCC. CEUS reliably detects minute vascularization in the periphery of this mostly necrotic tumor. [20] Due to higher spatial and temporal resolution and the ability to visualize minimal contrast enhancement, CEUS may detect higher Bosniak types compared to CT/MRI. [21–23]. Our study confirmed these findings: CEUS surpassed CT/MRI-Bosniak-types in 40.4%. Hence, CEUS-Bosniak and CT/MRI-Bosniak agreed fairly (κ = 0.28), as found by others. [24] The adverse impact of false positive CT/MRI-Bosniak registrations (especially IIF and III) is currently being addressed by additional application of CEUS. However, the distinction of predominantly cystic RCC from MCRNLMP remains a notorious challenge for imaging techniques. [25, 26] In our study, MCRNLMP (*n* = 7) was assigned to CEUS-Bosniak-III (3/7), and CEUS-Bosniak-IV (4/7) and to CT/MRI-Bosniak-IIF (1/7), and CT/MRI-Bosniak-III (6/7). In summary, one should not try to equalize Bosniak categories, despite morphological overlap.

Ultrasound follow-up allowed grading and the monitoring of the dynamics of cyst sizes. In only 1/105 cysts, the initial CEUS-Bosniak category changed during follow-up. All CEUS-Bosniak-IIF cysts in active surveillance remained stationary, and 29/30 CEUS-Bosniak-III lesions. In our cohort, we noted a change of cyst sizes in all Bosniak categories (enlargement and diminution, differences statistically not significant). The change in cyst sizes is probably mostly due to variations of cyst contents (i.e. blood). This is in line with observations in other studies, claiming that size change is not a useful discriminator of malignancy. Vascularity, solid areas, and thickened septa represent better indicators of malignancy. [8, 17].

**Fig. 3:**
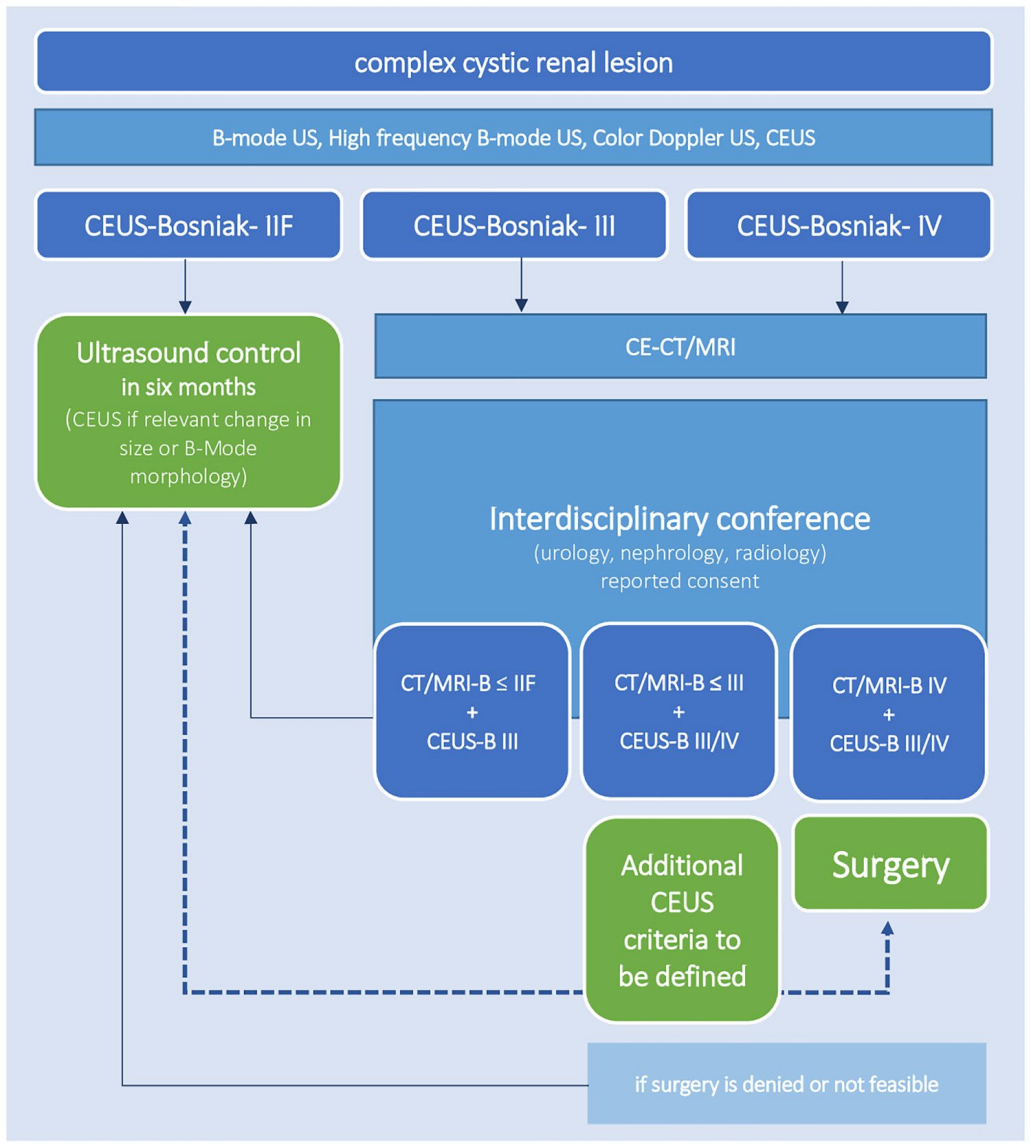
Proposal for a prospective multicenter CEUS-Bosniak register for complex cystic renal lesions

Finally, classification and appropriate treatment of complex cystic lesions should always be discussed in interdisciplinary boards (urologists, radiologists, internists, pathologists) to elaborate patient management. These imaging modalities in combination with individual evaluation of therapy options on the background of comorbidities represent the actual clinical management of renal cystic lesions (Fig. 3). On the basis of our findings, that suggest a potential major benefit of CEUS in this context, we suggest that CEUS should be the basis of future refinement of diagnostic criteria within this imaging system. A redefinition of CEUS-Bosniak classification will profit from a detailed documentation of CEUS findings and correlation with histopathology in a multicentric registry.

### Limitations

Short observation period, retrospective design, small single-institution cohort, small number of histopathologically evaluated lesions are limiting factors. The diagnostic power of each imaging modality (CT/MRI/CEUS) could not be calculated from our data.

Prospective, multicenter studies comparing CEUS with the current imaging standard are needed.
